# Normocomplementaemic urticarial vasculitis: effective treatment with omalizumab

**DOI:** 10.1186/s13601-018-0222-y

**Published:** 2018-09-21

**Authors:** Marianne de Brito, Gisela Huebner, DedeeF Murrell, Peter Bullpitt, Karin Hartmann

**Affiliations:** 1 Department of Dermatology, St George Hospital, University of New South Wales, Sydney, Australia; 2 Department of Dermatology, University of Luebeck, Luebeck, Germany; 3 University of Luebeck, Luebeck, Germany

**Keywords:** Chronic urticaria, Normocomplementaemic urticarial vasculitis, Omalizumab, Urticaria, Urticarial vasculitis

## Abstract

We report two patients with normocomplementaemic urticarial vasculitis with impressive response to omalizumab. This contrasts recent reports on hypocomplementaemic urticarial vasculitis syndrome, highlighting the need for clinical trials of omalizumab in normocomplementaemic urticarial vasculitis.

To the Editor:

Urticarial vasculitis is a cutaneous small vessel vasculitis characterised by erythematous patches or wheals persisting for greater than 24 h. Microscopic early appearances are of a neutrophilic leukocytoclastic vasculitis, which is thought to develop later into a lymphocytic vasculitis. It is associated with itching or burning, which can significantly reduce quality of life.

The presence of systemic features, involving the musculoskeletal, renal, pulmonary and/or gastrointestinal systems, is primarily linked to hypocomplementaemic urticarial vasculitis (HUV; syn. hypocomplementaemic urticarial vasculitis syndrome), for which anti-C1q auto-antibodies are a marker, and is increasingly seen as a separate entity to normocomplementaemic urticarial vasculitis (NUV).

Cutaneous symptoms of urticarial vasculitis are treated with oral antihistamines and can also require non-steroidal anti-inflammatory drugs, oral corticosteroids, colchicine, chloroquine and/or dapsone. Treatment is challenging due to the limited effect and side effects of current treatments.

Chronic spontaneous urticaria, where individual lesions persist for less than 24 h, has been shown to respond to omalizumab, a humanised, anti-IgE monoclonal antibody [1]. Clinically, urticarial vasculitis is often indistinguishable from chronic spontaneous urticaria unless lesion duration is carefully monitored, requiring histology for diagnosis, and may therefore be underdiagnosed. It could be extrapolated that amongst the chronic urticaria cases responding to omalizumab are also unrecognised cases of urticarial vasculitis.

There are only a few, isolated case reports of omalizumab being used in urticarial vasculitis with conflicting responses. Here, we present two cases of urticarial vasculitis with impressive therapeutic response immediately to a single dose of omalizumab.

A 63 year-old Australian male developed an urticarial rash in August 2016 after removing carpet in his house. Past medical history was significant for ischaemic heart disease, hypercholesterolaemia, hypertension and Barrett’s oesophagus. He was on several medications (no recent changes) and had no known allergies. On examination, there were widespread urticarial plaques and papules with signs of post-inflammatory hyperpigmentation, which persisted in the same areas over 24 h (Fig. 1a). He’d had an incomplete response to oral antihistamines (cetirizine 10 mg once daily and promethazine 12.5 mg twice daily) and to oral corticosteroids (prednisone 50 mg once daily with failure to taper below 15 mg/day over a month period) with ongoing pruritus continuing to affect his sleep.

**Fig. 1:**
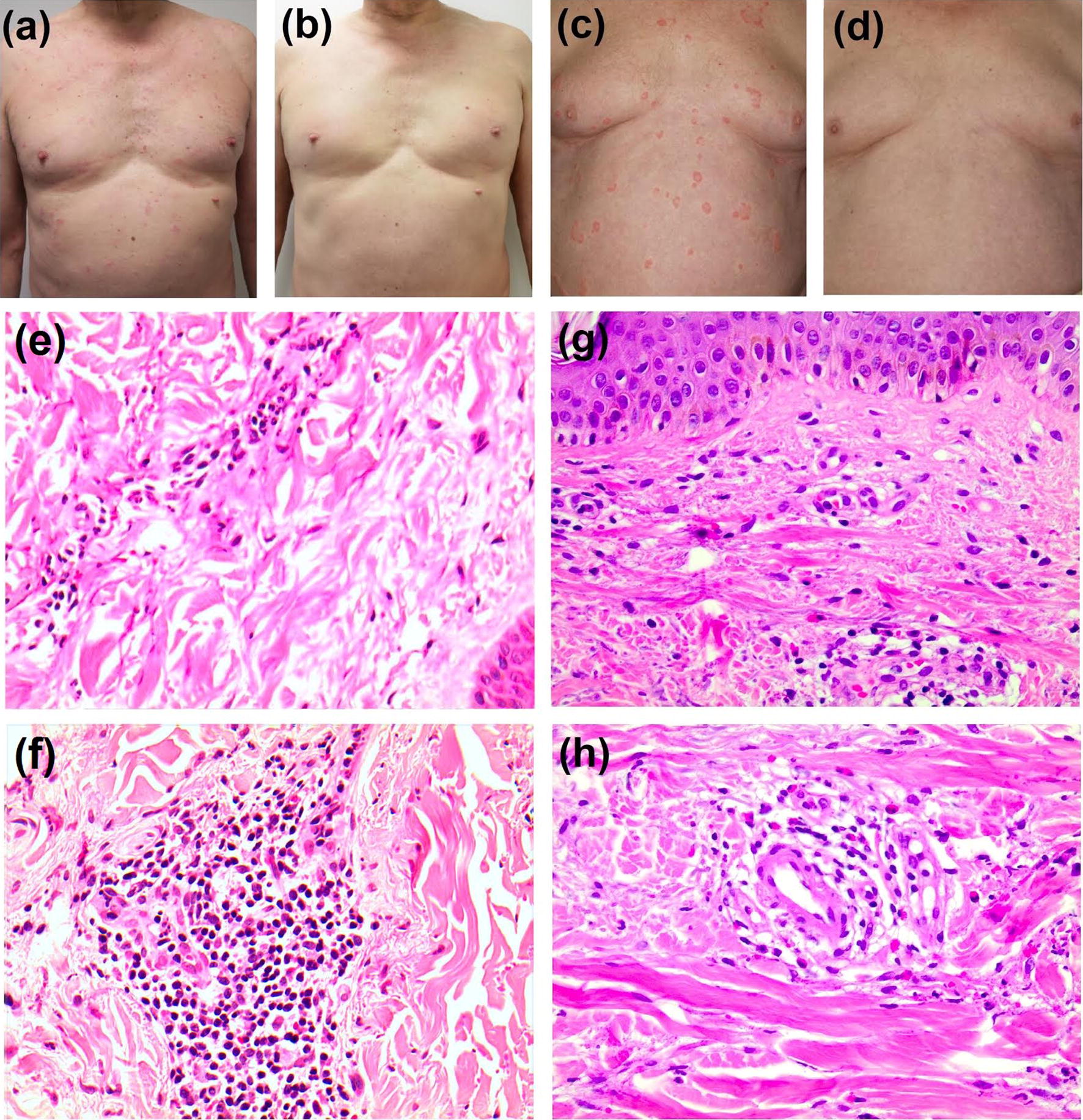
Patient 1—clinical photography of patient before (**a**) and after (**b**) treatment, histopathological sections of skin showing leukocytoclastic vasculitis with mild erythrocyte extravasation (**e**, **f**). Patient 2—clinical photography of patient before (**c**) and after (**d**) treatment, and histopathological sections of skin showing leukocytoclastic vasculitis with perivascular neutrophilic infiltrate and erythrocyte extravasation around small dermal vessels (**g**) and fibrinoid deposits within a vessel wall (**h**)

Routine blood tests including auto-antibodies, tumour markers and C3/C4 levels were normal, aside from likely steroid-induced leukocytosis and fasting hyperglycaemia. CT abdomen/pelvis revealed no significant findings. A skin biopsy revealed perivascular lymphocytes and neutrophils, with erythrocytic extravasation and leukocytoclasis, as demonstrated by a basophilic hue to the collagen surrounding vessels in some foci. These histological findings were consistent with urticarial vasculitis (Fig. 1e, f).

For the next 8 months, he had ongoing symptoms requiring high-dose systemic corticosteroids (cumulative dose 5460 mg prednisone with signs of glucocorticoid toxicity including raised BMI and impaired glucose tolerance), narrow band UVB therapy three times weekly, hydroxychloroquine (200 mg twice daily), colchicine (500 mcg twice daily) and sodium cromoglicate (100 mg once daily). In April 2017, he achieved a complete remission of his symptoms when he was started on omalizumab at a dose of 300 mg (150 mg × 2) subcutaneously once every four weeks. On review 4 weeks after the initial dose, his UAS7 (urticaria activity score over 7 days) had decreased from 30 to 0 and itch score had decreased from 9 to 0. Steroids and UV therapy were tapered down and stopped. To the present date (May 2018), he continues on omalizumab and remains symptom-free on omalizumab monotherapy.

The second case, a 72 year-old German male had a 7 year history of chronic wheals persisting over 24 h (Fig. 1c) and pronounced itching, which were characterised histologically as urticarial vasculitis (Fig. 1g, h). Investigations were normal including X-ray of the lungs and abdominal ultrasound, and there were no indications of extra-cutaneous symptoms or of other conditions responsible for causing urticarial vasculitis.

He had ongoing symptoms despite high dose desloratadine (5 mg QDS), dapsone (100 mg once daily) over 6 months, chloroquine (250 mg once daily) over 3 months and methotrexate (15 mg once weekly) over 6 weeks—stopped early due to deranged liver function. In November 2015, he was started on omalizumab monotherapy, also at 300 mg (150 mg × 2) subcutaneously once every 4 weeks. On review at 4 weeks, his UAS 7 had reduced from 42 to 1. Four weeks after a further dose, he was entirely symptom-free and continues treatment with omalizumab monotherapy at the same dose.

To our knowledge, there have been only a few case reports of omalizumab in urticarial vasculitis. Table 1 shows that some studies showed significant improvement in symptoms in male and female patients with NUV alone [2–6] and in combination with systemic lupus erythematosus [7] and Churg–Strauss syndrome [8]. However, studies in patients with HUV show conflicting results: one with major organ involvement showed only minimal improvement [9], whereas a patient without major organ involvement showed complete resolution. None of these studies reported adverse events in relation to omalizumab.

**Table 1:** Published cases of omalizumab use in urticarial vasculitis

References	Details	Age (years)	Complement levels	Systemic symptoms	Omalizumab		Effect of omalizumab
					Frequency (weeks)	Dose (mg)	
Del Pozo et al. [7]	Female with systemic lupus erythematosus (SLE)	51	Normal C3, low C4	Arthralgia (SLE)	Unknown, based on weight and IgE level		Partial resolution after 3 × doses
Varricchi et al. [8]	Female with Churg-Strauss syndrome (CSS)	44	Unknown	Asthma (CSS)	2	300	Complete, immediate resolution
Diez et al. [2]	Female	51	Normal	No	4	150	Complete resolution after 4–5 doses, relapse on cessation
	Female	54	Unknown	No	4	150	
	Female	28	Normal	No	4	300	Complete immediate resolution, relapse on cessation
Sussman et al. [3]	Unspecified adult		Unknown	Unknown	2	150	Complete resolution
Kai et al. [6]	Unspecified adult		Normal	No	4	150	Response, relapse on cessation
Ghazanfar et al. [4]	Male	68	Unknown	No	4	300	Complete resolution
Aurich et al. [9]	Female	36	Low C3 and C4	Nephrotic syndrome	4	300–600	No response over 19 months
Nucera et al. [10]	Female	47	Low C3 (0.17 g/L), normal C1q and anti C1q	No major organ dysfunction but arthralgia/abdominal pain	4	300	Complete resolution after 2nd injection. Complement levels returned to normal. Relapse on treatment cessation

Interestingly, these case reports suggest that omalizumab may be effective in NUV, rather than HUV. Omalizumab’s pharmacological mode of action is to block the effects of IgE on mast cells, hence reducing inflammation. Rather, HUV has been shown in case reports to respond to interleukin-1 receptor antagonism with anakinra or canakinumab. It would follow that IgE could play a pivotal role in the pathogenesis of NUV, but not in HUV with major organ involvement, and that the two disease categories could thus be related to different pathophysiological processes. It is possible that IgE and mast cells may be more central in the pathogenesis of NUV than vascular damage due to immune complex deposition, indicating that NUV is rather on the spectrum of chronic spontaneous urticaria. This would mean that the conventional histological definition of urticarial vasculitis may need revision. Although there may be a lymphocytic phase in the resolution of a leucocytoclastic vasculitis, there is no known evidence that neutrophilic leucocytoclastic vasculitis consistently develops into a lymphocytic vasculitis. These present cases in the context of the literature suggest that omalizumab is safe and effective in patients with normocomplementaemic urticarial vasculitis, but not hypocomplementaemic urticarial vasculitis syndrome. This sheds light on the distinct pathogenetic mechanisms of these two disease entities and may encourage clinical trials with omalizumab in normocomplementaemic urticarial vasculitis. Treatment of these patients with omalizumab would not least allow avoidance of glucocorticoid-induced toxicity.

## Abbreviations

CSS: Churg–Strauss syndrome
CT: computed tomography
HUV: hypocomplementaemic urticarial vasculitis
NUV: normocomplementaemic urticarial vasculitis
SLE: systemic lupus erythematosus
UAS: urticaria activity score
UV: ultraviolet
